# *Pharmaceuticals*: Impact Factor or CiteScore™

**DOI:** 10.3390/ph10030061

**Published:** 2017-07-06

**Authors:** Jean Jacques Vanden Eynde

**Affiliations:** Editor-in-chief, MDPI AG, St. Alban-Anlage 66, 4052 Basel, Switzerland; jean-jacques.vandeneynde@ex.umons.ac.be; Tel.: +32-2-355-81-61

June 14, 2017: announcement by Clarivate Analytics of the 2016 impact factors for scientific journals. For a few, this event is as important as the release of the Michelin red guide in France or the last installment of the Harry Potter series!

Indeed, recognition of a scientific journal can be evaluated by counting how many citations refer to articles published in the said journal. This idea led, in the mid-1950s, to the invention of the Journal Impact Factor by Eugene Garfield, founder of the Institute for Scientific Information® (ISI®) [1]. Later on, the idea was developed by ISI Thomson and Thomson Reuters Intellectual Property & Science division. The concept has become so popular that it obsesses some academic authorities, which strongly encourage their researchers to only publish in journals with high impact factors.

As quoted by E. Garfield [2]: “A journal’s impact factor is based on 2 elements: the numerator, which is the number of citations in the current year to items published in the previous 2 years, and the denominator, which is the number of substantive articles and reviews published in the same 2 years.” Therefore,

$${\mathrm{IF}}_{2016}=\frac{\mathrm{number of citations in}\;2016\;\mathrm{to articles published in}\;2015\;\mathrm{and}\;2014}{\mathrm{number of articles published in}\;2015\;\mathrm{and}\;2014}.$$

Web of Science™ (WoS™) is the data source used to calculate journal impact factors by Clarivate Analytics. It contains more than 33,000 journals [3] and the 2017 Journal Citation Reports announced that 11,459 journals have received a 2016 impact factor [4]. Amazingly, it is impossible to find in that volume a value for *Pharmaceuticals*, despite its encouraging development [5]. This is due to the fact that a first application for being considered in the Journal Citation Reports was rejected in 2014 and that Clarivate Analytics had informed MDPI AG that a second application could not be introduced before May 2017.

Fortunately for *Pharmaceuticals* and many other journals, a publisher, namely Elsevier, launched the concept of “CiteScore™” at the end of 2016 [6]. Utilizing the Scopus® database, which covers 20,346 journals, CiteScore™ is a novel metric reflecting the visibility of journals. Values are calculated in the same way as the journal impact factor of Clarivate Analytics, except that the previous three years are considered. Thus,

$${{\mathrm{CiteScore}}^{\mathrm{TM}}}_{2016}=\frac{\mathrm{number of citations in}\;2016\;\mathrm{to articles published in}\;2015,2014,\;\mathrm{and}\;2013}{\mathrm{number of articles published in}\;2015,2014,\;\mathrm{and}\;2013}.$$

Following Elsevier, the 2016 CiteScore™ of *Pharmaceuticals* has been estimated to be 4.90 [7]. Such an excellent score allows the journal to be considered the second best journal published by MDPI AG [8]. The value also indicates that *Pharmaceuticals* is now ranked #8/168 in the category Pharmaceutical Science and #21/158 in the category Molecular Medicine. This is wonderful news, rewarding the numerous efforts made by the members of the editorial offices and those of the editorial board, as well as the confidence of our readers, authors, and reviewers.
